# Rapamycin attenuates mitochondrial injury and renal tubular cell apoptosis in experimental contrast-induced acute kidney injury in rats

**DOI:** 10.1042/BSR20180876

**Published:** 2018-11-21

**Authors:** Xueyan Yang, Xiaojie Yan, Dingping Yang, Junke Zhou, Jie Song, Dingwei Yang

**Affiliations:** 1 Department of Nephrology, General Hospital of Tianjin Medical University, Tianjin 300052, China; 2 Department of Emergency, The Affiliated People’s Hospital of Zhangjiakou University, Hebei 075000, China; 3 Department of Nephrology, Renmin Hospital of Wuhan University, Wuhan, Hubei 430060, China; 4 Department of Nephrology, The Affiliated Hospital of Logistical College of Chinese People’s Armed Police Forces, Tianjin 300162, China; 5 Department of Nephrology, Tianjin Hospital, Tianjin 300211, China

**Keywords:** Acute renal injury, Apoptosis, Autophagy, Contrast media, mitochondrial injury, Oxidative stress

## Abstract

Reactive oxygen species (ROS) overproduction and renal tubular epithelial cell (TEC) apoptosis are key mechanisms of contrast-induced acute kidney injury (CI-AKI). Mitochondria are the main source of intracellular ROS. In the present study, the characteristics of mitophagy and the effects of rapamycin on contrast-induced abnormalities in oxidative stress, mitochondrial injury and mitophagy, TEC apoptosis and renal function were investigated in a CI-AKI rat model. Rats were divided into control group, CI-AKI group, and pretreatment groups (with rapamycin dose of 2 or 5 mg/kg). CI-AKI was induced by intraperitoneal injection of iohexol (12.25 g iodine/kg). Renal malondialdehyde (MDA) and catalase (CAT) were measured as oxidative markers. Light-chain 3 (LC3), P62, Beclin-1, PTEN-induced putative kinase (Pink1), and cytochrome *c* (Cyt *c*) expression were measured by Western blot. Mitochondrial membrane potential (ΔΨm) was determined by JC-1, colocalization of LC3-labeled autophagosomes with TOMM20-labeled mitochondria or LAMP2-labeled lysosomes was observed by fluorescence microscopy. Significantly increased serum creatinine (Scr), MDA and CAT, obvious mitochondrial injury including increase in cytosolic/mitochondrial Cyt *c* and decrease in ΔΨm, TEC apoptosis were induced by contrast administration. Contrast administration induced an increased expression of LC3II/I, Beclin-1, and Pink1 and decreased expression of P62. Rapamycin pretreatment induced overexpression of LC3II/I and Beclin-1. Moreover, LC3-labeled autophagosomes increasingly overlapped with TOMM20-labeled mitochondria and LAMP2-labeled lysosomes in CI-AKI, which was further enhanced by rapamycin administration. Contrast-induced Scr increase, oxidative stress, mitochondrial injury, TEC apoptosis, and necrosis were dose-dependently attenuated by rapamycin pretreatment. Rapamycin exerts renoprotective effects against CI-AKI by attenuating mitochondrial injury and oxidative stress, which might be associated with increasing mitophagy.

## Introduction

Contrast medium is used widely in many diagnostic and interventional procedures, and is a necessary component in modern medical technology. Unfortunately, this usage is linked to an increase in hospital-acquired acute renal failure as contrast-induced acute kidney injury (CI-AKI) becomes more and more common, especially considering the rate of growth of at-risk patient population [1,2]. The precise mechanisms underlying CI-AKI are not completely understood, especially its cellular and molecular mechanisms. Oxidative stress and renal tubular cell apoptosis are key mechanisms of CI-AKI [2–4]. However, the specific pathways of contrast-induced reactive oxygen species (ROS) overproduction and renal tubular epithelial cell (TEC) apoptosis are not clear.

Autophagy, a cellular process of degradation of damaged organelles, protein aggregates and other macromolecules in the cytoplasm, is considered to play important roles in cellular homeostasis [5,6]. It has been reported that suppressing autophagy could aggravate CI-AKI [7]. Furthermore, our previous *in vitro* studies have demonstrated that enhancing autophagy could attenuate contrast-induced TEC and podocyte cell apoptosis [8,9], which might be associated with alleviating oxidative stress. Mitochondria are known to be the main intracellular sites for ROS production. Damaged mitochondria will lead to the release of ROS and cytochrome *c* (Cyt *c*), followed by the induction of apoptosis [10]. Mitophagy, selective removal of damaged mitochondria, is expected as a means to protect cells from overshooting ROS production [11].

Rapamycin, a potent immune modulator, was originally used to treat transplant rejection and some autoimmune diseases. It has currently been found that rapamycin can enhance the expression of autophagy by inhibiting mammalian target of rapamycin (mTOR) pathway and help maintain normal cell metabolism [12]. In the present study, the pharmacological inducer of autophagy, rapamycin, was employed to investigate the effects of enhancing autophagy on contrast-induced abnormalities in mitophagy, mitochondrial injury, oxidative stress, TEC apoptosis, renal histopathology, and renal function.

## Materials and methods

### Ethics statement

The present study was approved by the Animal Care Committee of Tianjin Medical University General Hospital. And all the experimental procedures were performed according to the Institutional Animal Care and Use Committee of Tianjin Medical University General Hospital. All surgery was performed under sodium pentobarbital anesthesia, and all efforts were made to ameliorate animal suffering.

### Experimental materials

Iohexol (350 mg/ml iodine) was purchased from Yangtze River Pharmaceutical Group (Jiangsu, China). Rapamycin was obtained from AbMole Bioscience Incorporation (Shanghai, China). The malondialdehyde (MDA), catalase (CAT), and mitochondria isolation kits were from Nanjing Jiancheng Bioengineering Institute (Nanjing, China). Mitochondrial membrane potential assay kit with JC-1 was purchased from Beyotime Institute of Biotechnology (Jiangsu, China). The terminal deoxynucleotidyl transferase-mediated dTUP-biotin nick end-labeling (TUNEL) detection kit and BCA protein assay kit were obtained from Boster (Wuhan, China). Antibodies of light-chain 3 (LC3), P62, Cyt *c*, Beclin-1, PTEN-induced putative kinase (Pink1), TOMM20, LAMP2 were obtained from Sigma Chemical Company. Alexa Fluor 488– or 594–conjugated secondary antibodies was purchased from Molecular Probes (Carlsbad, CA). Sprague–Dawley rats were obtained from Beijing HFK Bioscience Co. Ltd (Beijing, China).

### Experimental design

Twenty four male Sprague–Dawley rats (8 weeks old; 210–220 g weight) were randomly assigned to control group (group Con, *n*=6), CI-AKI group (group CI-AKI, *n*=6), Rapamycin-pretreated groups (group Rap1 and group Rap2, each *n*=6). CI-AKI was induced by intraperitoneal injection of iohexol (12.25 g iodine/kg). Rapamycin with the final concentration of 2 mg/kg (group Rap1) or 5 mg/kg (group Rap2) was intraperitoneally administrated for 7 consecutive days before iohexol intraperitoneal injection. Rats in group Con were given an intraperitoneal injection of 0.9% saline. The dosages of iohexol used in the present study were determined based on the results of our preliminary study (data are shown in the preliminary study as Supplementary material). One day following contrast administration, all animals were anesthetized with sodium pentobarbital (30 mg/kg). Blood samples were drawn from the inner canthus vein and the kidneys were removed for the measurement of histopathology, apoptosis, autophagy, mitochondrial injury, MDA and CAT levels. The rats were killed by exsanguination. All rats were fed commercial chow and allowed free access to water in the present study.

### Measurement of serum creatinine and histopathological investigation

Serum creatinine (Scr) was measured with the picric acid method by the automatic biochemical analyzer. The fixed kidney tissue was dehydrated in ascending grades of ethanol and embedded in paraffin. Kidney tissue blocks were cut into 4-μm sections and subjected to Hematoxylin–Eosin (H.E.) staining. Tubular necrosis was graded as following [13]: no damage (− or 0), mild (± or 1, unicellular, patchy isolated damage), moderate (+ or 2, damage <25%), severe (++ or 3, damage between 25 and 50%), and very severe (+++ or 4, damage >50%).

### Measurement of renal MDA and CAT

Renal MDA levels were measured as described by our previous study [14] to evaluate the intensity of oxidative stress. Briefly, MDA was allowed to react with thiobarbituric acid, which yielded a red-colored product that was quantitated spectrophotometrically by measuring the absorbance at 532 nm. Renal CAT activity was determined according to the procedure for the CAT kit according to the manufacturer’s instructions.

### Mitochondria isolation

Purified mitochondria from kidney cells were separated using a mitochondrial extraction kit, according to the manufacturer’s instructions. Briefly, fresh kidney tissue was cut into pieces and homogenized in 1.0 ml cold Lysis Buffer. The homogenates were centrifuged at 1000×***g*** for 5 min at 4°C, the supernatant was centrifuged at 1000×***g*** for 5 min again. And then the supernatant was centrifuged at 12000×***g*** for 10 min at 4°C. The 12000×***g*** pellets were used to separate the cytosolic and mitochondrial fractions, of which, the sediments were mitochondrial fractions. The isolated mitochondrial or cytosolic fraction was collected for Western blot and mitochondrial membrane potential (ΔΨm) assay.

### Western blot analysis

Renal tissues and mitochondrial or cytosolic fractions were homogenized and lysed, followed by protein quantitation by the BCA method. The proteins were separated by SDS/PAGE (12% gel) and electrotransferred on to PVDF membranes. The blots were incubated with primary antibodies against LC3 (1:1000 dilution), P62 (1:1000 dilution), Beclin-1 (1:1000 dilution), Cyt *c* (1:1000 dilution), Pink1 (1:800 dilution) at 4°C overnight, respectively. β-actin (1:5000 dilution) was used as the loading control. After the membrane was washed three times in TBS + Tween-20 (TBST) for 10 min each, it was incubated with the HRP–conjugated secondary antibodies for 2 h at room temperature. Then the membrane was washed three times in TBST for 10 min again. The blotted protein bands were visualized by ECL Western blot detection reagents and exposed to X-ray film. The results were quantitated by the Quantity One Software (ImageJ) and β-actin was normalized to loading control.

### Determination of ΔΨm

ΔΨm was determined by the fluorescent probe, JC-1. Flow cytograms of JC-1 showed two distinct subpopulations with different ΔΨm, red with high ΔΨm and green with low ΔΨm. This shift in JC-1 fluorescence from red to green indicated a collapse of ΔΨm. The dissociated mitochondria suspension stained with 500 μl JC-1 in 5% CO_2_ incubator at 37°C for 20 min. The mixture was centrifugated at 2000 rpm for 5 min, and then the sediment was washed by Incubation Buffer. The fluorescence of JC-1 was recorded on a fluorescence microscope. The fluorescence intensity of red or green and the ratio of red/green were calculated and analyzed by Image-Pro Plus 6.0 software. The level of ΔΨm was reflected by the ratio of red/green.

### Immunofluorescence staining

After deparaffinization, serum blocking, and antigen retrieval, sections underwent double immunofluorescence staining with primary antibodies against LC3 (1:400 dilution) and TOMM20 (1:500) or LAMP2 (1:400) at 4°C overnight. Alexa Fluor 488– or 594–conjugated secondary antibodies were added to sections for 2 h at room temperature. TO-PRO-3 was used for nuclear counterstaining. Sections were mounted with a coverslip, sealed with nail polish, and stored in the dark at 4°C. And then sections were visualized using a laser scanning confocal microscope (400×, Fluoview FV1000, Olympus). Mander’s Overlap Coefficient was measured and analyzed as described by Image-Pro Plus 6.0 software [15]. Five randomly selected fields from one coverslip were included to get an average, and experiments were repeated independently at least three times.

### TUNEL staining using paraffin sections

TEC apoptosis was examined using TUNEL method. In brief, tissue sections were incubated with proteinase K for 10 min and then washed with PBS. Sections were respectively immersed in TUNEL reaction mixture which contained TdT and DIG-dUTP for 3 h at 37°C, anti-DIG-dUTP for 2 h at 37°C, and SABC-FITC + POD for 30 min at 37°C. For negative control, sections were incubated without TdT. And then sections were incubated with DAB followed by examination under microscope (Olympus BX53, Japan). TUNEL-positive renal tubular cells, as well as unstained cells, were counted at random in ten nonoverlapping fields (magnification: ×400). The average number of TUNEL-positive cells for each group was calculated for quantitation. The apoptotic index was calculated as the percentage of TUNEL-positive cells/total number of renal cells. All counting procedures were performed blindly.

### Statistical analysis

The software of SPSS Statistics 17.0 was employed to process the data. All data were expressed as the mean ± S.D. Multiple group comparison was performed using ANOVA, followed by Bonferroni or Dunnett post-hoc tests. Histological data were analyzed by the Kruskal–Wallis nonparametric tests and Steel-type multiple comparison tests. Statistical significance was defined as *P*<0.05.

## Results

### Renal function

As shown in Table 1, the level of Scr increased notably after contrast administration (Con compared with CI-AKI, *P*<0.05), achieved the general standard for CI-AKI. Pretreatment with high-dose rapamycin significantly attenuated the contrast-induced increase in Scr (Rap2 compared with CI-AKI, *P*<0.05).

**Table 1:** Changes in renal function, MDA, and CAT levels 1 day following contrast administration with or without rapamycin pretreatment

	Group Con (*n*=6)	Group CI-AKI (*n*=6)	Group Rap1 (*n*=6)	Group Rap2 (*n*=6)
Scr (μmol/l)	50.92 ± 13.32	232.65 ± 34.00^1^	206.32 ± 45.67^1^	156.94 ± 16.34^1,2,3^
MDA (nmol/g tissue)	2.11 ± 0.41	11.23 ± 1.58^1^	5.63 ± 1.03^1,2^	3.78 ± 0.84^1,2,3^
CAT (μ/g tissue)	4.61 ± 0.66	18.06 ± 1.78^1^	10.53 ± 1.23^1,2^	8.07 ± 1.19^1,2,3^

Data are presented as mean ± S.D. Descriptions of the groups are in the text. ^1^*P*<0.05 compared with Con. ^2^*P*<0.05 compared with CI-AKI. ^3^*P*<0.05 compared with Rap1.

### Renal histopathology

**Figure 1:**
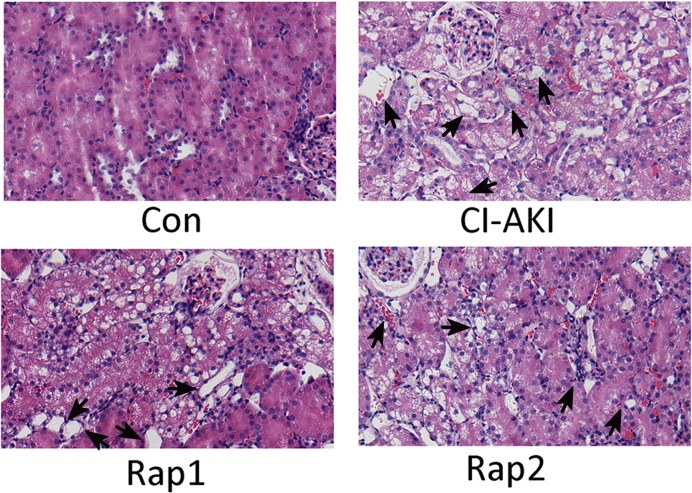
Light microscopy of renal tissue of rats in group Con (Con), group CI-AKI (CI-AKI), group Rap1 (Rap1), and group Rap2 (Rap2) Descriptions of the groups are in the text. Intraperitoneal injection of large dose of contrast media induced severe renal tubular degeneration and necrosis (group CI-AKI). Rapamycin markedly and dose-dependently attenuated contrast media-induced renal tubular degeneration and necrosis (group Rap1 and Rap2). Arrows indicate tubular necrosis and cell denudation (Hematoxylin-and-Eosin stain; magnification: ×200). Each group (*n*=6).

As shown in Figure 1, the severe renal tubular cell necrosis and cell denudation were noted in the cortex and outer stripe of the medulla in the rats of CI-AKI group. Pretreatment with high-dose rapamycin significantly alleviated the contrast-induced renal tubular necrosis. The grade of histopathological changes is depicted in Table 2.

**Table 2:** Histopathological changes in kidneys of normal and CI-AKI rats with or without rapamycin pretreatment

Tubular necrosis					
Histopathological	−	±	+	++	+++
Changes/grade	0	1	2	3	4
Group Con (*n*=6)	6	0	0	0	0
	(0.00 ± 0.00)				
Group CI-AKI (*n*=6)	0	0	0	2	4
	(3.60 ± 0.52^1^)				
Group Rap1 (*n*=6)	0	0	0	4	2
	(3.33 ± 0.52^1^)				
Group Rap2 (*n*=6)	0	2	3	1	0
	(1.83 ± 0.75^1,2,3^)				

Data are expressed as number of animals with corresponding histopathological changes. Values in parentheses represent the mean ± S.D. of histopathological changes/grade. Descriptions of the groups are in the text. Grades: no damage (− or 0), mild (± or 1), moderate (+ or 2), severe (++ or 3), and very severe (+++ or 4). ^1^*P*<0.05 compared with Con. ^2^P<0.05 compared with CI-AKI. ^3^*P*<0.05 compared with Rap1.

### Oxidative stress marker

Oxidative stress, as reflected by the degree of lipid peroxidation measured by MDA and CAT assay, was significantly increased after contrast administration (Con compared with CI-AKI, *P*<0.05; Table 1). The kidney MDA and CAT levels dramatically and dose-dependently decreased in rats of group Rap1 and Rap2 compared with that of group CI-AKI.

### The autophagy-related protein expression

**Figure 2:**
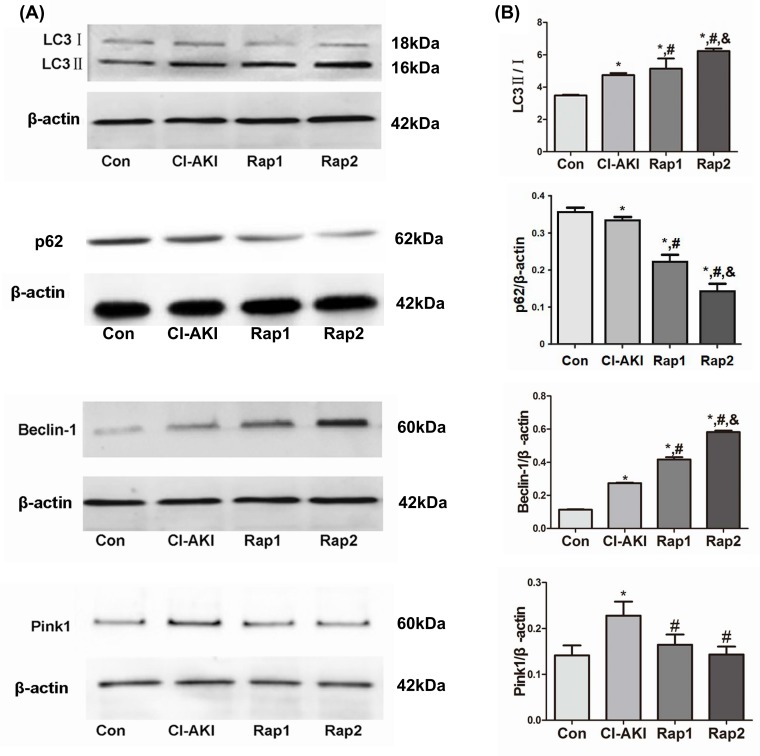
Changes in autophagy- and mitophagy-related protein expression following contrast exposure with or without rapamycin (**A**) The expression of LC3, P62, Beclin-1, and Pink1 protein was measured by Western blot analysis in kidneys from rats in group Con (Con), group CI-AKI (CI-AKI), group Rap1 (Rap1), and group Rap2 (Rap2). Descriptions of the groups are in the text. Contrast exposure induced autophagy in TEC cells. Rapamycin significantly up-regulated the expression of LC3-II/I, Beclin-1 and down-regulated the expression of P62. (**B**) Data show the mean ± S.D. Experiments were repeated three times. *P<0.05 compared with Con; ^#^*P*<0.05 compared with CI-AKI; ^&^*P*<0.05 compared with Rap1.

Autophagy is a highly regulated process by numerous molecules, in which LC3 and P62 are recognized as an autophagosome marker. The formation of autophagosome involves the conversion of LC3 from LC3I into LC3II and the subsequent combination with P62. The increase in LC3II/I ratio and the down-regulation of P62 expression can be used as a hallmark of up-regulated autophagy [11,16]. Another critical molecule, Beclin-1, a component of the phosphatidylinositol 3-kinase (PI3K) complex, has a key role in the early stage of formation of autophagosome membranes [17]. Pink1- and Parkin-mediated mitophagy is currently the most well-established mechanism mediating mitophagy in mammalian cells [18]. Currently, Pink1 overexpression and subsequent Parkin translocation to mitochondria and Parkin-dependent ubiquitination of mitochondrial proteins are often used to show activation of mitophagy [19]. In the present study, the expression of LC3-II/I, Beclin-1, and Pink1 protein significantly up-regulated and the expression of P62 significantly down-regulated in the rats in group CI-AKI compared with those in group Con (Figure 2), which demonstrated that autophagy and mitophagy were activated by contrast exposure. Rapamycin pretreatment induced the up-regulation of LC3II/I and Beclin-1 and the down-regulation of P62, which showed that rapamycin enhanced the autophagy.

### Mitochondrial injury

**Figure 3:**
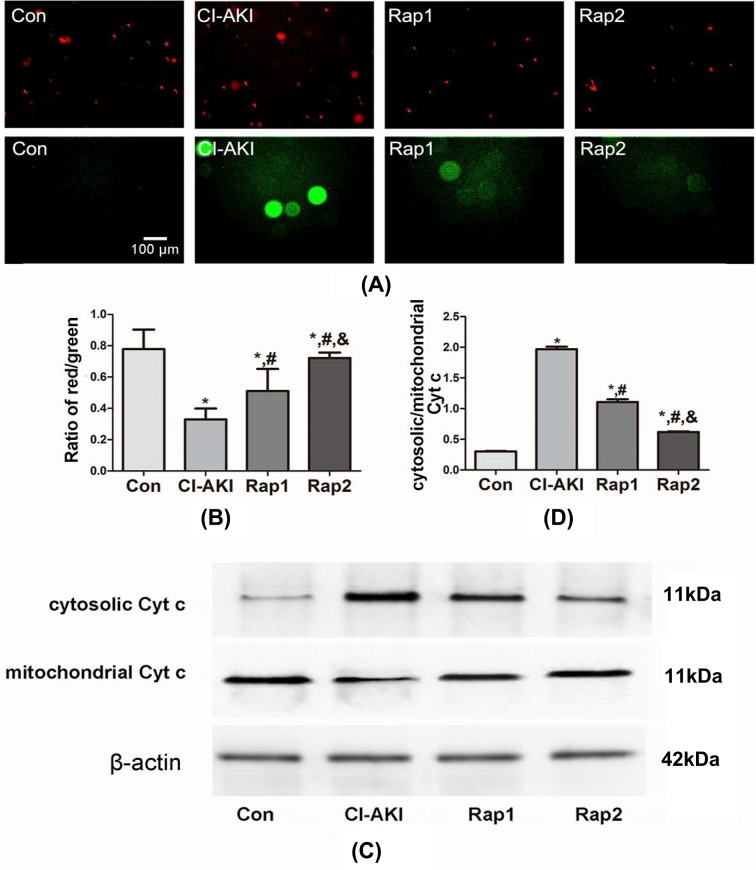
Changes in mitochondrial injury following contrast exposure with or without rapamycin (**A**) ΔΨm in kidneys from rats in group Con (Con), group CI-AKI (CI-AKI), group Rap1 (Rap1), and group Rap2 (Rap2) was detected by JC-1. Descriptions of the groups are in the text. Contrast exposure induced the significant decrease in ΔΨm. Rapamycin pretreatment alleviated the decline of ΔΨm. (**B**) Columns represent the ratio of red/green. The fluorescence intensity of red or green and the ratio of red/green were calculated and analyzed by Image-Pro Plus 6.0 software. Data show the mean ± S.D. Experiments were repeated three times. (**C**) The expression of Cyt *c* in cytoplasm and mitochondria from kidneys in group Con (Con), group CI-AKI (CI-AKI), group Rap1 (Rap1), and group Rap2 (Rap2) was measured by Western blot analysis. Descriptions of the groups are in the text. The ratio of cytosolic/mitochondrial Cyt *c* obviously increased following contrast exposure. Rapamycin pretreatment significantly attenuated contrast-induced the release of Cyt *c* from the mitochondria. (**D**) Columns represent the gray-scale value of Cyt *c* expression. Data show the mean ± S.D. Experiments were repeated three times. *P<0.05 compared with Con; ^#^*P*<0.05 compared with CI-AKI; ^&^*P*<0.05 compared with Rap1.

ΔΨm, as estimated using JC-1, has provided a valuable indicator of cells’ health and functional status [20]. As shown in Figure 3A,B, JC-1 staining showed a decrease in ΔΨm in rats of group CI-AKI which had a lower ratio of red/green (Con compared with CI-AKI, *P*<0.05). Furthermore, the ratio of red/green was significantly and dose-dependently increased following rapamycin pretreatment (Rap1/2 compared with CI-AKI, *P*<0.05; Rap2 compared with Rap1, *P*<0.05), which demonstrated that rapamycin has protective effects on contrast-induced mitochondrial injury. Recent studies also have shown that release of mitochondrial Cyt *c* is a marker of mitochondrial injury and a critical step in the apoptosis process [21,22]. As shown in Figure 3C,D, increased cytosolic/mitochondrial Cyt *c* was observed in the rats of group CI-AKI (Con compared with CI-AKI, *P*<0.05). Pretreatment with rapamycin obviously and dose-dependently suppressed the release of contrast-induced mitochondrial Cyt *c*, which indicated that rapamycin protects against contrast-induced mitochondrial injury (Rap1/2 compared with CI-AKI, *P*<0.05; Rap2 compared with Rap1, *P*<0.05).

### Double-immunofluorescence analysis

**Figure 4:**
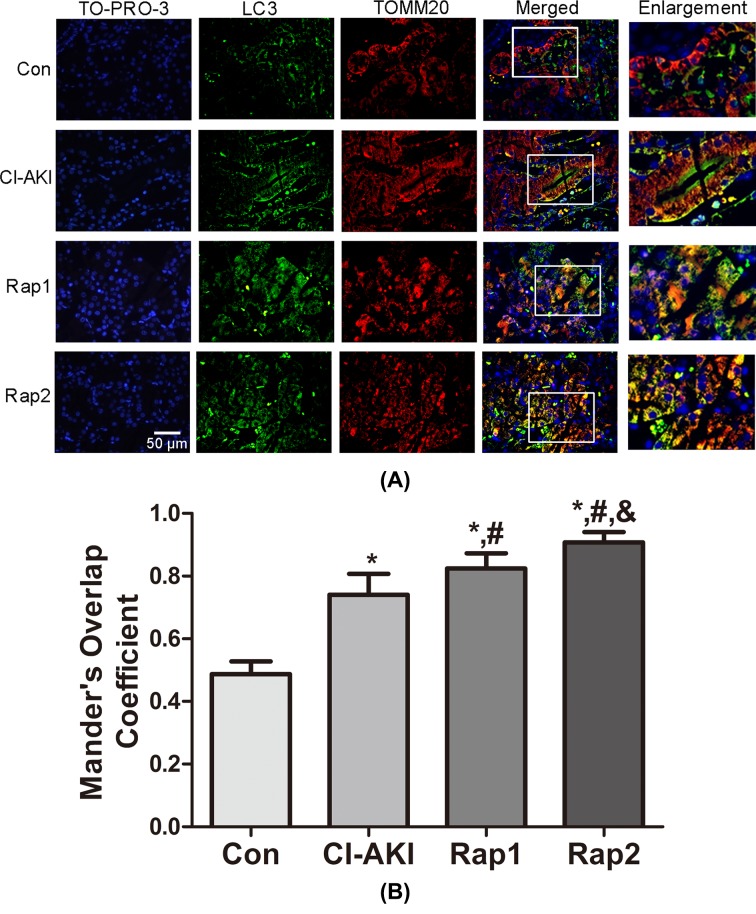
Immunoflouresence-double labeling of LC3 with TOMM20 following contrast exposure with or without rapamycin (**A**) Kidney sections from rats in group Con (Con), group CI-AKI (CI-AKI), group Rap1 (Rap1), and group Rap2 (Rap2) were double immunofluorescence stained for LC3-labeled autophagosomes (green) and TOMM20-labeled mitochondria (red). Nuclei were stained with TO-PRO-3 (blue). Areas in the white boxes are enlarged at right. Descriptions of the groups are in the text. Strong colocalization signals of LC3-labeled autophagosome (green) and TOMM20-labeled mitochondria (red) in group CI-AKI were seen as yellow-orange dots on merging, suggesting the presence of mitophagy. Moreover, TOMM20-labeled mitochondria increasingly overlapped with LC3-labeled autophagosomes with rapamycin pretreatment. (**B**) Columns represent Mander’s overlap coefficient. At least 60 cells from three independent experiments for each group were included. Data are expressed as mean ± S.D. *P<0.05 compared with Con; ^#^*P*<0.05 compared with CI-AKI; ^&^*P*<0.05 compared with Rap1.

**Figure 5:**
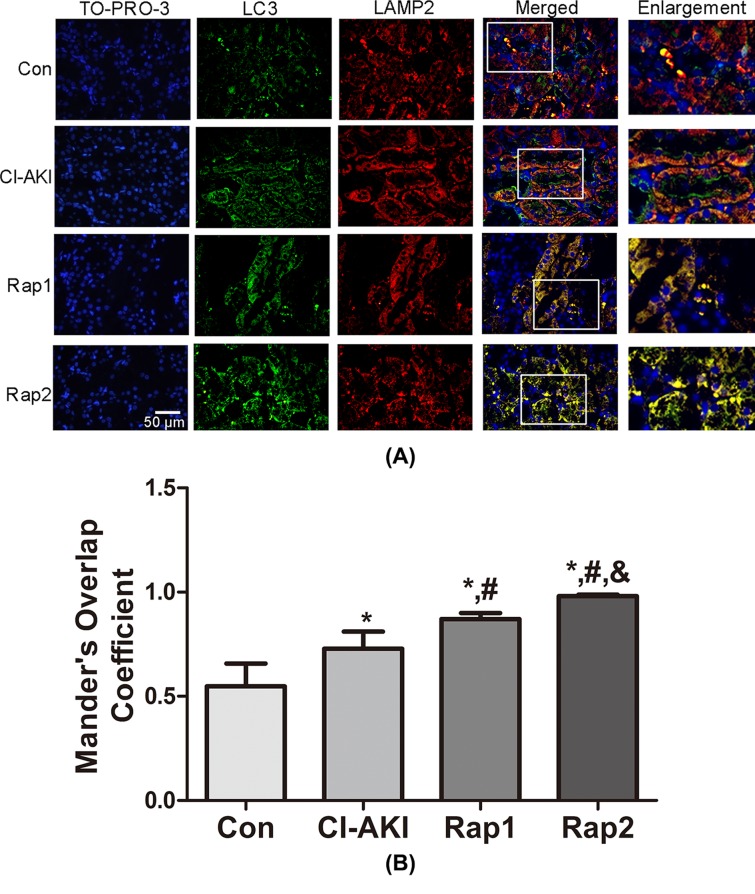
Immunoflouresence double labeling of LC3 with LAMP2 following contrast exposure with or without rapamycin (**A**) Kidney sections from rats in group Con (Con), group CI-AKI (CI-AKI), group Rap1 (Rap1), and group Rap2 (Rap2) were double immunofluorescence stained for LC3-labeled autophagosomes (green) and LAMP2-labeled lysosomes (red). Nuclei were stained with TO-PRO-3 (blue). Areas in the white boxes are enlarged at right. Descriptions of the groups are in the text. Colocalization signals of LC3-labeled autophagosome (green) and LAMP2-labeled lysosome (red) increased in group CI-AKI, which were further enhanced following rapamycin administration. (**B**) Columns represent Mander’s overlap coefficient. At least 60 cells from three independent experiments for each group were included. Data are expressed as mean ± S.D. *P<0.05 compared with Con; ^#^*P*<0.05 compared with CI-AKI; ^&^*P*<0.05 compared with Rap1.

To further confirm the autophagic degradation of mitochondria (mitophagy) in response to contrast exposure, colocalization of TOMM20-stained mitochondria and LC3-stained autophagosomes was performed in renal tissue. As shown in Figure 4, contrast treatment markedly enhanced LC3 dot formation with concomitant colocalization with TOMM20-stained mitochondria in group CI-AKI, indicating contrast-induced mitophagy activation. Moreover, the overlap was significantly and dose-dependently enhanced following rapamycin pretreatment, which demonstrated that rapamycin enhanced the mitophagy. The same results could be observed in colocalization of LC3-stained autophagosomes and LAMP2-stained lysosomes (Figure 5), indicating increased autophagic activity.

### TEC apoptosis

**Figure 6:**
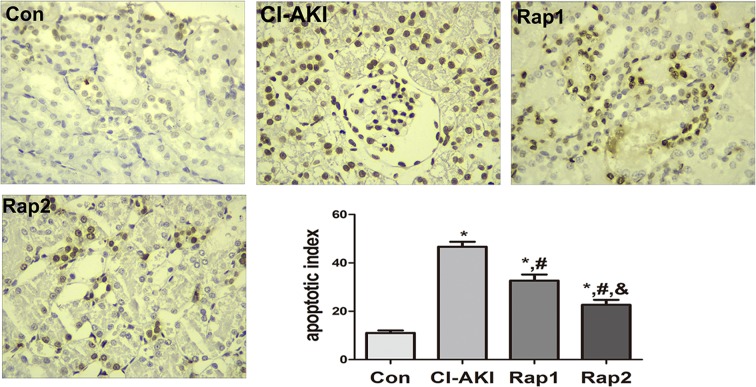
Changes in renal TEC apoptosis following contrast exposure with or without rapamycin Renal TEC apoptosis in group Con (Con), group CI-AKI (CI-AKI), group Rap1 (Rap1) and group Rap2 (Rap2). Descriptions of the groups are in the text. Contrast exposure induced significantly increased apoptosis in renal TEC (group CI-AKI). Rapamycin obviously and dose-dependently alleviated contrast-induced renal tubular apoptosis (group Rap1, group Rap2). (TUNEL; magnification: ×400). Columns showed the apoptotic index in rats of group Con (Con), group CI-AKI (CI-AKI), group Rap1 (Rap1), and group Rap2 (Rap2). *P<0.05 compared with Con; ^#^*P*<0.05 compared with CI-AKI; ^&^*P*<0.05 compared with Rap1. Each group (*n*=6).

As shown in Figure 6, significantly increased TEC apoptosis was induced by contrast administration (Con compared with CI-AKI, *P*<0.05). Pretreatment with rapamycin significantly and dose-dependently attenuated contrast-induced TEC apoptosis. The TEC apoptotic index in rats of group Rap1 and Rap2 was markedly lower than that of group CI-AKI (Rap1/2 compared with CI-AKI, *P*<0.05; Rap1 compared with Rap2, *P*<0.05).

## Discussion

CI-AKI is broadly defined as a decrease (25%) in glomerular filtration rate or a certain absolute (44 μmol/l) or relative (25%) increase in Scr after contrast administration within 2–3 days in the absence of an alternative etiology [2,23]. In the present study, large dose of contrast intraperitoneal injection induced significant increase in Scr. Histopathological analyses also showed that large dose of intraperitoneal contrast administration induced severe tubular necrosis. These data clearly demonstrated that CI-AKI was successfully induced by intraperitoneal injection of large dose of contrast. Compared with the doses of 5.25, 8.75 g I/kg (data are shown in Supplementary material), it was larger dose (12.25 g I/kg) of contrast media with the same osmolality and viscosity that induced CI-AKI, which indirectly suggested that the nephrotoxicity of contrast media was associated with the dose of contrast media.

Oxidative stress has been verified to play a crucial role in the mechanisms of CI-AKI, and the antioxidants have been a major focus in the research about CI-AKI prevention [24–26]. But the mechanism how contrast induced ROS overproduction remains unknown. Mitochondria as the powerhouse of the cell are the major sites of ATP production, but at the same time they generate ROS. Large quantities of ROS might be released from the mitochondria when they are insulted and injured. Whether contrast exposure would result in mitochondrial injury and subsequent ROS overproduction remains unclear. In the present study, contrast administration induced significant increase in Cyt *c* of the cytosol and obvious decrease in ΔΨm, which verified that contrast administration can induce severe mitochondrial injury. Significantly increased renal MDA and CAT level were also observed in rats of group CI-AKI, which implied that contrast administration induced oxidative stress or ROS overproduction. Taken together, these data showed that mitochondrial injury and oxidative stress were involved in the development of CI-AKI.

Autophagy, an evolutionarily conserved mechanism, plays a significant role in maintaining cell homeostasis and adaptation to various stress situations including nutrient limitation, oxidative stress, hypoxia, and accumulation of protein aggregates or damaged organelles [27]. There are two well-characterized signaling cascades that sense nutrient status and extracellular or intracellular stress, the mTOR and AMP-activated protein kinase (AMPK) pathways, in the regulation of autophagy [28]. mTOR is a serine/threonine kinase that accepts the interaction of a variety of upstream signaling, such as Class I PI3K, IGF-1/2, and MAPK, and was considered to be an inhibitor of autophagy. mTOR has two kinds of different complex forms, one is mTORC1 which is sensitive to rapamycin, one is mTORC2 which is not sensitive to rapamycin. The former mainly regulate cell growth, cell apoptosis, energy metabolism, and cell autophagy, while the latter is mainly related to cytoskeleton recombination and cell survival [29]. AMPK can activate autophagy via two independent mechanisms: suppression of mTORC1 activity and direct control of ULK1 phosphorylation [30]. It has been reported that enhancing autophagy protects podocyte from contrast exposure by the means of reducing oxidative stress [8], but its detailed mechanisms remain unclear. The present results demonstrated that up-regulated expression for LC3-II/I, Beclin-1 and decreased expression of P62 were observed in the kidneys in rats of group CI-AKI compared with control rats, which showed that contrast administration induced autophagy. Furthermore, rapamycin pretreatment induced the increased expression of LC3-II/I and Beclin-1 and decreased expression of P62 compared with rats in group CI-AKI without rapamycin, which showed that rapamycin enhanced autophagy. In addition, this was confirmed by colocalization of the autophagosomal compartment (LC3) and lysosomal compartment (LAMP2) using double-labeling immunofluorescence (Figure 5). Rapamycin administration significantly attenuated the contrast-induced increase in Cyt *c* release from the mitochondria and the decrease in ΔΨm, which suggested that enhancing autophagy alleviated the contrast-induced mitochondrial injury. Significant decreases in Scr, MDA, and CAT level were also observed in rats in group Rap2 compared with that of group CI-AKI. Histopathological observation found that rapamycin administration ameliorated contrast-induced TEC necrosis. All these data demonstrated that rapamycin alleviated contrast-induced mitochondrial injury and oxidative stress and exerted protective effects on CI-AKI.

Damaged or dysfunctional mitochondria can be cleared from the cells by mitophagy [18,31]. Mitophagy is activated by mitochondrial membrane depolarization, followed by overexpression of Pink1 on mitochondrial outer membrane, which then recruits an E3 ubiquitin ligase (Parkin) from the cytosol [18,32]. This initiates the localization of damaged mitochondria to membranes containing microtubule-associated protein LC3 leading to the formation of autophagosomes [12]. These autophagosomes containing the damaged mitochondria fuse with lysosomes which are cleared from the cells [18,31,32]. Accumulation of damaged or dysfunctional mitochondria, due to impaired mitophagy or mitophagy inadequacy, has been associated with many pathologic conditions [33,34]. In order to investigate whether mitophagy is involved in the pathogenesis of CI-AKI, the expression of Pink1 and the double-labeling immunoflurescence with LC3 and TOMM20 were examined. As we know, the damaged mitochondria can induce the overexpression of Pink1 and activate the mitophagy [18,32]. In the present study, contrast exposure induced severe mitochondrial injury and the Pink1 expression significantly increased in rats in group CI-AKI compared with control rats (Figure 2), which demonstrated that contrast exposure activated the mitophagy. Results of double-labeling immunoflurescence with LC3 and TOMM20 showed that rapamycin enhanced the integration of autophagosome and damaged mitochondria, which suggested that rapamycin further enhanced mitophagy. But, Western blot results showed that rapamycin pretreatment did not up-regulate, but down-regulate the contrast-induced overexpression of Pink1. In our opinion, the more severe the mitochondrial damage, the stronger the expression of PINK1. The reason why the overexpression of Pink1 induced by contrast was down-regulated by rapamycin might be that rapamycin pre-treatment alleviated contrast-induced mitochondrial injury. Another possibility might be that the balance of autophagy and mitophagy may vary over time. Based on the facts that rapamycin up-regulated the expression of LC3 and Beclin-1 but not Pink1, and the fact that rapamycin alleviated the contrast-induced mitochondrial injury and ROS overproduction, we can hypothesize that rapamycin should be able to enhance the integration of recognized damaged mitochondria with membranes containing LC3 and accelerate the formation of autophagosomes and the clearance of damaged mitochondria.

**Figure 7:**
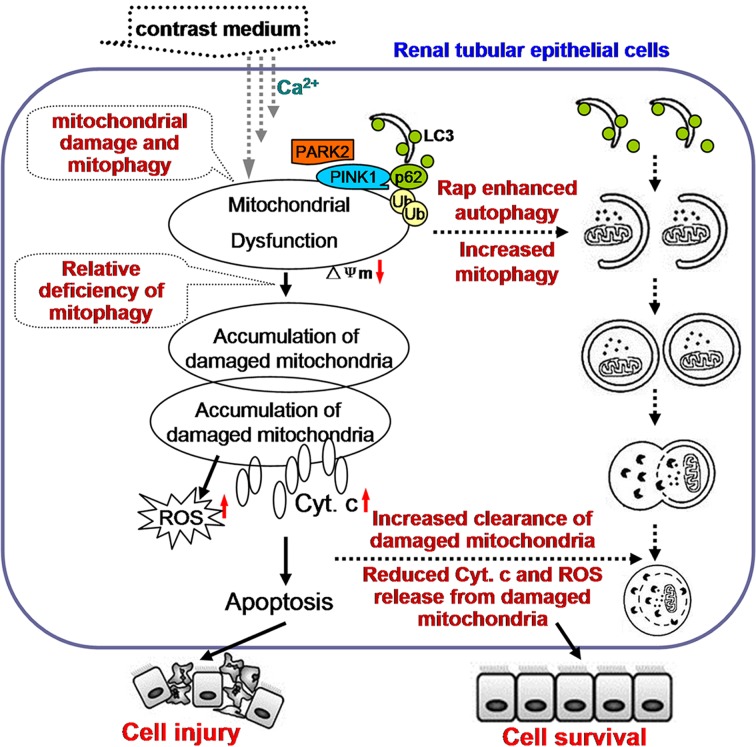
The hypothesis that the relative inadequency of autophagy and mitophagy is involved in contrast-induced TEC apoptosis and CI-AKI Contrast exposure causes mitochondrial injury (possibly through calcium influx), which activates autophagy and mitophagy. But, the activated autophagy and mitophagy are relatively inadequate for the large quantities of damaged mitochondria. So large quantities of damaged mitochondria accumulated in cytoplasm, which can further induce ROS and Cyt *c* release from the mitochondria and result in TEC apoptosis and CI-AKI. Enhancing autophagy help integrate the damaged mitochondria with membranes containing LC3 and accelerate the clearance of damaged mitochondria, which further alleviates the oxidative stress and ameliorates contrast-induced TEC apoptosis and CI-AKI.

TEC apoptosis and TEC apoptosis is the key mechanism of CI-AKI. Our previous studies have shown that ROS overproduction and endoplasmic reticulum stress are implicated in contrast-induced TEC apoptosis and that ROS generation might be an event ahead of endoplasmic reticulum stress in the iopromide-induced apoptosis [35]. In the present study, contrast exposure induced a significant increase in TEC apoptosis, and it was ameliorated by rapamycin pretreatment (Figure 6). As discussed above, rapamycin pretreatment alleviated contrast-induced mitochondrial injury and suppressed the contrast-induced oxidative stress, which intimates that the damaged mitochondria might be one of the sources of ROS. Based on these results, we proposed the possible mechanism that contrast exposure induce TEC apoptosis and the possible cause that rapamycin alleviated CI-AKI. Contrast exposure results in mitochondrial injury, which might be ascribed to calcium influx [36], induces the overexpression of Pink1 and activates autophagy and mitophagy in order to clear the damaged or dysfunctional mitochondria. But, the activated autophagy and mitophagy are relatively inadequent for the large quantities of damaged mitochondria. This resulted in accumulation of damaged or dysfunctional mitochondria, which can further induce ROS and Cyt *c* release from the mitochondria and result in TEC apoptosis and CI-AKI. Rapamycin enhanced autophagy and helped intergrate the damaged mitochondria with membranes containing LC3 and accelerate the clearance of damaged mitochondria, which further alleviates the oxidative stress and ameliorates contrast-induced TEC apoptosis and CI-AKI. This hypothesis was shown in Figure 7.

## Conclusion

Rapamycin exerts renoprotective effects against contrast-induced TEC apoptosis and CI-AKI by suppressing mitochondrial injury and subsequent ROS overproduction, which might be associated with increasing mitophagy. This suggested that autophagy and mitophagy regulation could be a promising way in prophylaxis and treatment against CI-AKI in clinical practice in the future.

## Supporting information

**Table S1:** Changes in renal function one day following contrast or saline administration (n=6).

## Abbreviations

AMPK: AMP-activated protein kinase
CAT: catalase
CI-AKI: contrast-induced acute kidney injury
Cyt *c*: cytochrome *c*
LC3: light-chain 3
MDA: malondialdehyde
mTOR: mammalian target of rapamycin
Pink1: PTEN-induced putative kinase
PI3K: phosphatidylinositol 3-kinase
PTEN: phosphatase and tensin homology
ROS: reactive oxygen species
Scr: serum creatinine
TBST: TBS + Tween-20
TEC: tubular epithelial cell
TUNEL: terminal deoxynucleotidyl transferase-mediated dTUP-biotin nick end-labeling
Δψm: mitochondrial membrane potential
