# Supplementary material for: Rapamycin attenuates mitochondrial injury and renal tubular cell apoptosis in experimental contrast-induced acute kidney injury in rats
Source: Biosci Rep. 2018 Nov 21;38(6):BSR20180876. doi: 10.1042/BSR20180876 (PMC6246763; doi:10.1042/BSR20180876)
Supplement: Supplementary file 1 [file bsr20180876_Supp1.pdf]

Preliminary study

Objective: To explore the animal model of contrast-induced acute kidney injury (CI-AKI), iohexol was injected by different ways with different dosage and renal function was measured one day following contrast administration.

Methods: 78 healthy Sprague-Dawley rats weighing of 210-230 g were randomly divided into thirteen groups (each n=6). The rats of each group were treated as follows. group A: tail vein injection group with 0.9% saline at the dose of 35 ml/kg, group B1-3: tail vein injection groups with iohexol at the dose of 3.5, 5.25, 8.75 g I/kg , group C1-3: intraperitoneal injection groups with iohexol at the dose of 5.25, 8.75, 12.25 g I/kg, group D1-2: two consecutive tail vein injection groups with iohexol at the dose of 5.25, 7.0 g I/kg, and group E1-4 (unilateral nephrectomy groups): uninephrectomized rats followed by tail vein injection of iohexol at the dose of 2.1, 2.8, 3.5, 5.25g I/kg. One day following contrast or saline administration, all animals were anesthetized with sodium pentobarbital (30 mg/kg). Blood samples were drawn from the inner canthus vein for the measurement of serum creatinine. All rats were fed commercial chow and allowed free access to water.

Results: As shown in S1 Table, compared with group A, increased serum creatinine was induced by contrast intraperitoneal injection with the largest dose in group C3. No significant increase in serum creatinine was observed in rats of other groups compared to group A.

Conclusion: The animal model of CI-AKI can be successfully induced by intraperitoneal injection of iohexol at the dose of 12.25 g I/kg.

Table 1 Changes in renal function one day following contrast or saline administration (n=6).

	Scr (umol/l)
group A	45.19±4.62
group B1	51.53±5.67
group B2	52.94±3.15
group B3	56.74±4.03
group C1	47.27±3.51
group C2	47.69±1.31
group C3	165.197±49.28[a]
group D1	55.33±0.45
group D2	54.89±1.16
group E1	44.65±6.80
group E2	51.93±5.68
group E3	45.16±4.00
group E4	51.55±4.45

Date are presented as mean ± SD. Scr： serum creatinine； [a]$P< 0.05$ vs group A; group A: tail vein injection group with 0.9% saline at the dose of 35 ml/kg; group B1-3: tail vein injection groups with iohexol at the dose of 3.5, 5.25, 8.75 g I/kg; group C1-3: intraperitoneal injection groups with iohexol at the dose of 5.25, 8.75, 12.25 g I/kg; group D1-2: two consecutive tail vein injection groups with iohexol at the dose of 5.25, 7.0 g I/kg; group E1-4: uninephrectomized rats followed by tail vein injection of iohexol at the dose of 2.1, 2.8, 3.5, 5.25g I/kg.
